# Inter-Arm Blood Pressure Differences and Handgrip Strength in Youth Athletes: Implications for Early Cardiovascular Screening

**DOI:** 10.3390/healthcare14111500

**Published:** 2026-05-28

**Authors:** Garyfallia Pepera, Chrysanthi Piperopoulou, Eleni Karagianni

**Affiliations:** Clinical Exercise Physiology and Rehabilitation Research Laboratory, Department of Physiotherapy, Faculty of Health Sciences, University of Thessaly, 35132 Lamia, Greece; cpiperopoulou@uth.gr (C.P.); elkaragiann@uth.gr (E.K.)

**Keywords:** inter-arm blood pressure difference, handgrip strength, children, adolescents, athletes

## Abstract

**Highlights:**

**What are the main findings?**

- Inter-arm SBP differences (≥10 mmHg) were present in 12.2% of youth athletes, supporting the importance of bilateral BP assessment.
- HGS was positively associated with SBP—particularly in boys—and both were higher in the dominant limb, while anthropometric factors strongly predicted systolic BP.

**What are the implications of the main findings?**

- Bilateral BP measurement may be considered as part of routine assessment in paediatric populations to improve early detection of cardiovascular risk.
- Simple, non-invasive tools such as HGS assessment combined with anthropometric monitoring can enhance early cardiovascular screening and understanding of physiological adaptations in youth athletes.

**Abstract:**

**Background:** Inter-arm blood pressure difference (IAD) ≥ 10 mmHg is an established marker of cardiovascular risk in adults; however, evidence in paediatric populations remains limited. Handgrip strength (HGS) is increasingly recognized as a simple and reliable indicator of neuromuscular fitness and cardiometabolic health during growth and development. **Objectives:** We aimed to investigate the association between IAD and HGS in children and adolescent athletes and to explore the influence of anthropometric characteristics. **Methods:** Forty-one non-professional athletes aged 6–16 years participated in this cross-sectional study. Anthropometric characteristics, bilateral resting BP, and bilateral HGS were assessed using standardized protocols. Inter-arm differences in systolic and diastolic BP were calculated. **Results:** An inter-arm systolic BP difference ≥ 10 mmHg was observed in 12.2% of participants. Dominant-arm systolic BP and HGS were significantly higher compared with the non-dominant side (*p* < 0.05). HGS showed a positive association with systolic BP, particularly in boys, whereas weaker associations were observed with diastolic BP. In regression analysis, BMI emerged as a significant predictor of systolic blood pressure. **Conclusions:** In young athletes, dominant-arm superiority in both muscular strength and systolic BP was evident. Bilateral BP assessment combined with HGS measurement may provide information during cardiovascular evaluation paediatric athletic populations.

## 1. Introduction

Elevated blood pressure (BP) during childhood and adolescence is increasingly recognized as a critical predictor of cardiovascular disease (CVD) risk later in life. Longitudinal observational cohorts have demonstrated that BP measured in early life tracks into adulthood, contributing to the development of sustained hypertension, target-organ damage, and adverse cardiovascular outcomes [1,2,3]. Also, recent epidemiological evidence indicates that hypertension is becoming increasingly common in children and adolescence, influenced by obesity, low physical activity, poor sleep quality, high sodium intake, as well as hereditary factors [4]. Consequently, accurate identification of abnormal BP patterns in paediatric populations is central to primordial prevention and early intervention strategies.

Accurate BP measurement is fundamental for diagnosing hypertension and stratifying cardiovascular risk in children. Current clinical guidelines for paediatric BP emphasize standardized measurement techniques, including appropriate cuff size, adequate rest before measurement, multiple readings, and measurement in a seated position [5]. Despite these recommendations, BP is frequently measured unilaterally in many clinical and field settings, which may overlook important physiological variation between limbs [6].

Inter-arm blood pressure difference (IAD) refers to discrepancies in systolic or diastolic BP between the left and right upper limbs. In adults, clinically significant inter-arm systolic differences (≥10 mmHg) have been associated with subclinical peripheral arterial disease, increased cardiovascular events, and all-cause mortality [7,8]. Recent meta-analytic evidence suggests that an inter-arm systolic BP difference ≥ 15 mmHg is a stronger predictor of cardiovascular mortality than smaller differences and may be particularly informative in younger adults [7]. Although large IADs have well-recognized prognostic significance in adult populations, fewer studies have systematically examined IAD in paediatric cohorts.

Emerging paediatric evidence indicates that IADs are present in children and adolescents, with a notable proportion exhibiting differences ≥ 10 mmHg when both arms are measured [9]. Such differences may lead to misclassification of BP status when measurements are performed unilaterally, underscoring the importance of bilateral BP measurement for accurate cardiovascular risk evaluation in pediatric populations.

During childhood and adolescence, the cardiovascular system undergoes significant maturation, influenced by growth, body size, vascular structural development, autonomic regulation, and pubertal hormonal changes [10]. Additionally, modifiable lifestyle factors such as physical activity levels, sedentary behavior, and body composition have been linked to variations in BP among youth [11,12]. For example, normative cardiovascular responses to exercise and recovery in young athletes vary by age, sex, and training status, underscoring the influence of sport participation on cardiovascular physiology [11].

Muscular strength is another key aspect of paediatric physical fitness with recognized associations to cardiometabolic health. HGS is widely used as a reliable surrogate measure of overall muscular strength and functional capacity due to its simplicity, reproducibility, and strong correlations with muscle mass and general fitness [13,14].

Although many studies have shown that high HGS is associated with lower systolic and diastolic BP in adults [15], the relationship between muscular strength and BP during youth remains complex and, at times, contradictory. Some paediatric studies report positive associations between greater HGS and elevated BP, suggesting that stronger muscle implies greater lean body mass and associated hemodynamic load (e.g., higher cardiac output), whereas others find little or no direct association or even inverse relationships when controlling for body composition [16,17]. These discrepancies likely reflect confounding effects of height, muscle mass, adiposity, maturational status, and physical activity patterns, underscoring the need to consider multiple interacting factors when interpreting strength–BP relationships in children and adolescents.

Youth athletes represent a physiologically distinctive subgroup in which regular participation in organized sports induces adaptations across multiple systems, including enhanced cardiovascular performance, improved vascular function, and increased muscle strength. Regular athletic training has been associated with beneficial cardiometabolic profiles and reduced cardiovascular risk factors in young populations [18]. Nonetheless, sport-specific training, especially in unilateral or asymmetrical disciplines, can lead to functional asymmetries between limbs that may extend beyond muscular strength to influence peripheral vascular properties [19].

Despite these considerations, relatively few studies have investigated whether asymmetries in muscular strength (e.g., HGS differences between hands) are associated with IADs in paediatric athletic cohorts. Understanding these relationships could elucidate physiological adaptations during growth and training, and inform screening strategies for early cardiovascular risk in children and adolescents.

Therefore, the primary aim of the present study was to investigate the associations between IAD and HGS in non-professional youth athletes aged 6–16 years. A secondary aim was to examine the influence of anthropometric characteristics on both BP and muscular strength in this population.

## 2. Materials and Methods

### 2.1. Study Design

This cross-sectional study was conducted in accordance with the ethical principles of the Declaration of Helsinki. Ethical approval was obtained from the Internal Ethics and Deontology Committee of the Department of Physiotherapy, University of Thessaly (protocol no. 18687/25/ΤΦΣΚΘ, 26 June 2025). Permission to conduct the study within the training facilities was also obtained from the collaborating sports academies. Written informed consent was obtained from the parents or legal guardians of all participants prior to data collection after full explanation of the study procedures, confidentiality measures, and the voluntary nature of participation. Verbal or written assent was obtained from children older than seven years when appropriate.

### 2.2. Participants

A convenience sample of youth athletes was recruited from two local sports academies: a rhythmic gymnastics and dance academy for girls and a basketball academy for boys. Initially, 44 children aged between six and sixteen years volunteered to participate, and three participants were excluded due to incomplete measurements, resulting in a final sample of 41 participants (16 girls and 25 boys). Inclusion criteria included age between six and sixteen years, regular participation in organized training within the sports academy, absence of known cardiovascular, metabolic, or musculoskeletal disorders, willingness to participate, and written parental consent. Exclusion criteria included injury or illness at the time of assessment, withdrawal from the study, incomplete data collection, or lack of parental consent.

### 2.3. Data Collection Procedures

All measurements were conducted during regular afternoon training sessions at the participants’ training facilities to minimize disruption to their routine activities. Data collection occurred between 27 June and 27 July 2025. Due to scheduling constraints within the sports academies, it was not possible for all evaluations to be conducted on the same day for every participant. However, to ensure maximum measurement accuracy and minimize temporal variability, efforts were made to complete all assessments for each child within a two-week period. The variables recorded included age, sex, dominant hand, anthropometric characteristics (height, body weight, and waist circumference), BP, and HGS. BP and HGS were assessed bilaterally for both upper limbs.

#### 2.3.1. Determination of Dominant Upper Limb

HGS was assessed following the protocol of the American Society of Hand Therapists (ASHT), according to which testing begins with the dominant upper limb. Therefore, identifying the participant’s dominant hand was essential. Dominance was determined by asking two questions: (a) which hand the participant uses for writing and (b) which hand is used more frequently in their sport, adopting the approach of Gasior et al. (2020) [20]. In cases where the two answers were inconsistent, participants were asked to indicate which hand they perceived as stronger.

#### 2.3.2. Blood Pressure Assessment

BP was measured using an automated upper-arm oscillometric sphygmomanometer (Microlife BP B3 AFIB, Microlife AG, St. Gallen, Switzerland) equipped with an appropriately sized pediatric cuff with a circumference of 18–22 cm. Prior to measurement, participants were informed about the brief discomfort they might feel during cuff inflation, as younger children may be unfamiliar with the procedure. They were also instructed to remain silent during the measurement to avoid fluctuations in BP values. Participants were seated comfortably with back support, feet flat on the floor, and both arms supported at heart level, so that the mean point of the cuff in the measured arm is aligned with the level of the right atrium, following the recommendations of the European Society of Hypertension for out-of-office BP measurements [21]. After a rest period of at least three minutes, measurements were obtained alternately from both arms. Three measurements were recorded for each arm with at least one minute between successive measurements to minimize variability. The mean value of the three measurements for each arm was used for analysis. IAD was calculated as the absolute difference between right- and left-arm systolic and diastolic BP values.

It is worth noting that for children whose feet did not reach the floor, a firm shoebox was used as a footrest to maintain proper posture. Similarly, when the arm was positioned higher than heart level, a chair cushion was placed under the elbow of the measured arm, to ensure correct alignment. These adjustments ensured standardized positioning for all participants regardless of height.

#### 2.3.3. Anthropometric Measurements

Body height was measured to the nearest 0.1 cm using a Leicester stadiometer (Seca Ltd., Birmingham, UK). Specifically, participants were instructed to remove their shoes and stand upright with heels together and their back aligned against the wall to ensure correct posture during height assessment. Body weight was recorded to the nearest 0.1 kg using a calibrated digital scale (TANITA BC-418 MA, Tanita Corporation, Tokyo, Japan). Waist circumference was measured using a non-elastic tape at the midpoint between the lowest rib and the iliac crest at the end of normal expiration. The tape was placed horizontally and in light contact with the skin, following World Health Organization (WHO) recommendations [22], while a detailed explanation of the procedure was provided to ensure children’s comfort and cooperation. Body mass index (BMI) was calculated as weight in kilograms divided by height in meters squared (kg/m^2^) and categorized according to age- and sex-specific WHO growth reference standards [23].

#### 2.3.4. Handgrip Strength Assessment

HGS was assessed using a Jamar hydraulic hand dynamometer (Sammons Preston Rolyan, Bolingbrook, IL, USA) following the standardized protocol recommended by the ASHT [24]. Participants were seated on an armless chair, maintaining an upright posture without leaning against the backrest, with hips and knees positioned at approximately 90°, and their feet placed flat and uncrossed on the floor. The tested arm was positioned with the shoulder adducted and neutrally rotated, the elbow flexed at 90°, the forearm in a neutral position, and the wrist in slight extension. Before the assessment, participants were instructed to maintain a steady body position throughout the test. To minimise unintentional upper limb movement during maximal effort, a second researcher gently supported the participant’s elbow to prevent compensatory motions. The dynamometer was set to the second handle position, which is considered the most comfortable and reliable setting for paediatric populations, and it’s also recommended by the ASHT. Each participant was encouraged to perform three maximal isometric contractions for each hand, beginning with the dominant hand, stopping immediately if they experienced any discomfort or pain. Verbal encouragement was provided to standardize motivation, and a minimum rest period of 15 s was allowed between trials to minimize fatigue. The mean value of the three trials for each hand was calculated to two decimal places for improved precision and was used for statistical analysis.

Given the young age of the participants, a brief familiarization procedure preceded the actual test. This included a demonstration of the task, instructions on how to properly grip the device, and one light practise trial at roughly 15% of their perceived maximum effort. The formal assessment began 1 to 2 min after the familiarisation trail to ensure adequate understanding of the procedure.

### 2.4. Statistical Analysis

Statistical analyses were performed using IBM SPSS Statistics version 29.0 (IBM Corp., Armonk, NY, USA), and statistical significance was set at *p* < 0.05. Descriptive statistics were calculated for all variables and are presented as means ± standard deviations. Analyses were conducted for the total sample and stratified by sex. The normality of data distribution was assessed using the Shapiro–Wilk test, in combination with visual inspection of histograms and Q–Q plots, as well as evaluation of skewness and kurtosis indices. Paired comparisons between the left and right upper limbs were performed to examine potential inter-arm differences in HGS, systolic blood pressure (SBP), and diastolic blood pressure (DBP). Depending on data distribution, paired *t*-tests or Wilcoxon signed-rank tests were applied. Correlation analyses were conducted to examine associations between HGS and BP measurements within each limb (left HGS with left SBP/DBP and right HGS with right SBP/DBP), and Pearson or Spearman correlation coefficients were calculated as appropriate. Additional analyses examined associations between inter-arm differences in BP (ΔSBP, ΔDBP) and inter-limb differences in HGS (ΔHGS). Inter-arm BP differences (ΔSBP and ΔDBP) were calculated as directional differences between right and left arm measurements (right-left). Absolute inter-arm differences were additionally used to identify clinically relevant systolic IAD ≥ 10 mmHg.

Finally, multiple linear regression analyses were performed to evaluate the predictive value of HGS and anthropometric variables (age, BMI, waist circumference, sex, and limb dominance) on systolic and diastolic BP values for each arm. For multivariable regression analysis, right-arm SBP was selected as the dependent variable, as it demonstrated the strongest associations with HGS in preliminary analyses. To maintain model parsimony and avoid multiple parallel outcome models, left-arm BP and DBP measures were not included in the regression analyses. Multicollinearity was assessed using variance inflation factor (VIF) and tolerance statistics. VIF values < 5 were considered acceptable.

## 3. Results

### 3.1. Participant Characteristics

A total of 41 children (16 girls, 25 boys) were enrolled in the study. The mean age of girls was significantly lower than that of boys (9.63 ± 2.25 vs. 11.96 ± 0.89 years, *p* < 0.001), suggesting a younger cohort among female participants. Boys were significantly taller (156.4 ± 9.1 vs. 139.2 ± 12.5 cm, *p* < 0.001) and heavier (52.2 ± 11.1 vs. 35.3 ± 10.3 kg, *p* < 0.001), resulting in a higher BMI (21.1 ± 2.8 vs. 17.8 ± 2.6 kg/m^2^, *p* = 0.002) and greater waist circumference (73.2 ± 6.6 vs. 63.0 ± 5.3 cm, *p* < 0.001).

The distribution of BMI categories revealed that most girls were of normal weight (68.8%), whereas boys showed higher prevalence of overweight (32%) and obesity (24%), indicating sex-specific differences in body composition. These anthropometric details are presented in Table 1, which also provides a baseline for interpreting functional measures such as BP and HGS.

These findings underscore the importance of accounting for sex and body composition when analysing paediatric functional outcomes, particularly strength and cardiovascular parameters.

### 3.2. Blood Pressure

Both SBP and DBP measurements were collected from the left and right arms. Mean SBP was slightly lower in girls than boys, though differences were not statistically significant (left arm: 107.0 ± 11.0 vs. 109.1 ± 11.7 mmHg, *p* = 0.411; right arm: 109.0 ± 9.6 vs. 110.6 ± 10.0 mmHg, *p* = 0.502). Similarly, DBP did not differ significantly between sexes (left arm: 66.1 ± 7.6 vs. 66.7 ± 7.2 mmHg, *p* = 0.786; right arm: 66.6 ± 6.5 vs. 66.5 ± 6.5 mmHg, *p* = 0.968) (Table 2).

**Figure 1:**
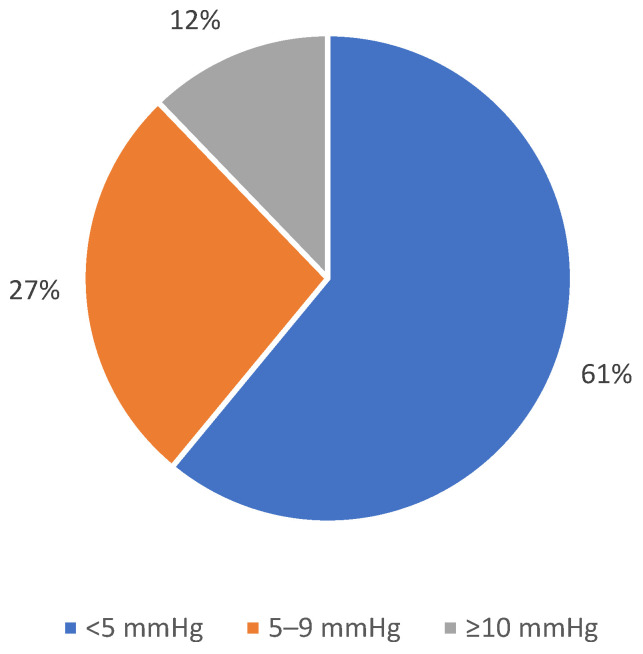
Distribution of inter-arm SBP differences among participants.

Directional inter-arm differences (right − left) were generally small in both sexes, with ΔSBP of 2.0 ± 1.6 mmHg in girls versus 1.5 ± 1.7 mmHg in boys (*p* = 0.290), and ΔDBP of 0.5 ± 0.7 mmHg in girls versus −0.2 ± 0.9 mmHg in boys (*p* = 0.073) (Table 2). An absolute ΔSBP ≥ 10 mmHg was observed in five participants (12.2% of the total sample). Given the small sample size, this prevalence estimate should be interpreted as exploratory and with limited precision. This finding suggests that clinically relevant IAD may be detectable even in youth athletes (Figure 1). These results suggest a relatively symmetrical distribution of BP between limbs in both sexes, consistent with normative paediatric cardiovascular physiology.

### 3.3. Hand Grip Strength

HGS showed clear sex-specific differences, particularly in inter-limb asymmetry, while it is important to highlight that 92.68% of participants were right-handed, as all girls and 22 of 25 boys were right-hand-dominant. Absolute HGS values were higher in boys for both left (20.7 ± 6.8 vs. 17.9 ± 5.3 kg, *p* = 0.171) and right hand (22.1 ± 6.8 vs. 19.2 ± 5.6 kg, *p* = 0.149), though these differences did not reach statistical significance, likely due to the smaller female sample size (Table 2).

However, ΔHGS was significantly greater in boys (2.6 ± 1.7 kg) compared to girls (1.3 ± 1.2 kg, *p* = 0.009), indicating more pronounced lateralization in male participants (Table 2). This finding aligns with known developmental patterns, where boys tend to demonstrate earlier onset of muscular lateralization.

### 3.4. Correlation Analysis

Correlation analysis demonstrated moderate positive associations between HGS and SBP in the total sample, particularly in the dominant limb. Right-hand HGS was moderately correlated with right-arm SBP (r = 0.548, *p* < 0.001) and weakly with DBP (r = 0.316, *p* = 0.044), whereas left-hand HGS showed a moderate association with left-arm SBP (r = 0.457, *p* = 0.003) but no significant relationship with DBP (r = 0.244, *p* = 0.124).

**Figure 2:**
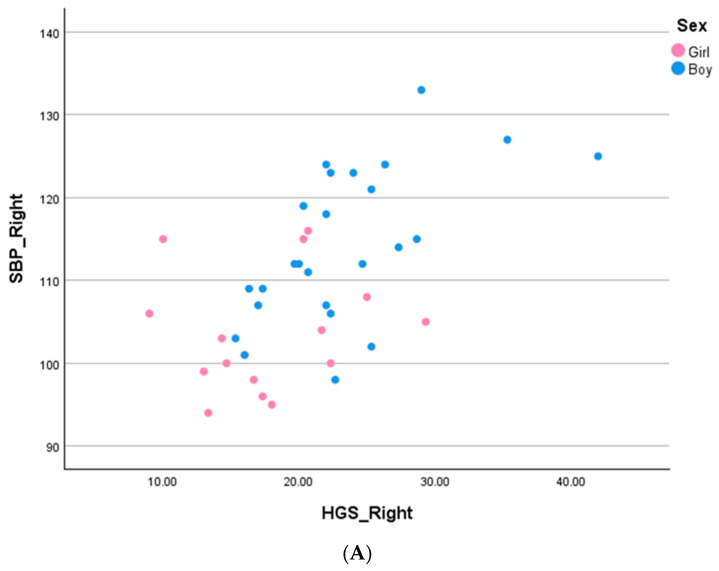

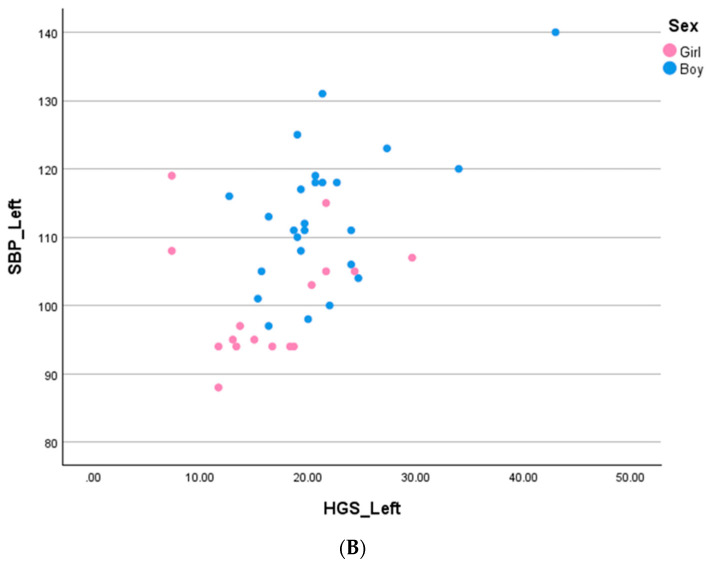
(**A**) Association between left-hand HGS and left-arm SBP in boys and girls. (**B**) Association between right-hand HGS and right-arm SBP in boys and girls.

Sex-stratified analyses revealed a clear divergence between boys and girls. In boys, right-hand HGS was moderately associated with both SBP (r = 0.596, *p* = 0.002) and DBP (r = 0.514, *p* = 0.009), while the association with left-arm SBP approached but did not reach statistical significance (r = 0.365, *p* = 0.073). In contrast, no significant associations were observed in girls (for *p* < 0.05), indicating sex-specific differences in the relationship between muscular strength and BP (Table 3) (Figure 2A,B). These findings suggest that the relationship between muscular strength and BP may be influenced by sex and body composition.

Overall, these results highlight sex-specific trends: boys demonstrate higher absolute strength and greater inter-limb asymmetry, while BP remains comparable between sexes. These findings are critical for the interpretation of paediatric functional assessments, informing both clinical evaluation and targeted intervention strategies.

### 3.5. Multiple Regression Analysis

In the multiple linear regression model for right-arm SBP, BMI emerged as the only significant independent predictor (B = 1.423, SE = 0.462, *p* = 0.004), suggesting that higher BMI was associated with higher SBP.

HGS was not independently associated with SBP after adjustment for anthropometric and demographic variables (B = 0.457, SE = 0.280, *p* = 0.111), indicating that the observed univariate associations were likely influenced by body size.

Age (B = −0.506, *p* = 0.600) and sex (B = 4.675, *p* = 0.124) were not statistically significant predictors in the adjusted model (Table 4). The regression model explained 56.4% of the variance in right-arm SBP (adjusted R^2^ = 0.515) (Table 5).

All predictors demonstrated low multicollinearity (VIF range: 1.12–2.03). Residual analysis and diagnostic testing indicated no major violations of linearity, normality, homoscedasticity, or multicollinearity assumptions.

## 4. Discussion

Although youth athletes are generally considered a healthy population, early identification of BP asymmetries and cardiovascular markers during childhood may contribute to a better understanding of early cardiovascular regulation and support preventive strategies targeting future hypertension and cardiometabolic disease. Within this context, exercise-based assessment approaches may provide clinically relevant information regarding early cardiovascular adaptation.

The present study investigated the association between IAD and HGS in youth athletes, while accounting for anthropometric characteristics. The main findings indicate that: (a) a clinically relevant systolic IAD (≥10 mmHg) was present in five participants (12.2%), (b) HGS was moderately associated with SBP, particularly in the dominant limb, (c) these associations were evident primarily in boys but not in girls, and (d) BMI emerged as the only independent predictor of SBP, whereas HGS was not retained in the adjusted model.

These findings expand current knowledge on cardiovascular asymmetry and muscular fitness in paediatric athletic populations and reinforce the importance of multidimensional assessment approaches combining hemodynamic and neuromuscular indicators.

From a clinical and preventive perspective, the presence of inter-arm systolic BP differences in a subset of physically active youth may have relevance for early cardiovascular monitoring. Although the observed prevalence should be interpreted cautiously due to the small sample size, such asymmetries may reflect early vascular or hemodynamic variability during growth. In the context of exercise-based preventive medicine, these findings highlight the importance of routine BP assessment in youth populations, particularly in sports settings, where physiological adaptations and asymmetries may emerge during development. Longitudinal studies are needed to determine whether early inter-arm differences persist over time and whether they are associated with future cardiometabolic risk trajectories.

### 4.1. Inter-Arm Blood Pressure Differences in Youth

The prevalence of systolic IAD observed in this study (12.2%) is consistent with recent evidence suggesting that inter-arm differences ≥ 10 mmHg occur in a non-negligible proportion of both adult and paediatric populations [25]. While IAD has traditionally been considered a marker of vascular dysfunction and subclinical atherosclerosis in adults, recent studies indicate that it may also reflect early vascular heterogeneity rather than overt pathology [26,27].

The greater variability observed in SBP compared to DBP aligns with current understanding that systolic pressure is more sensitive to arterial stiffness, stroke volume, and wave reflection phenomena [28,29,30]. In young athletic populations, these differences may additionally reflect training-induced vascular adaptations and asymmetrical limb loading.

Importantly, even in a physically active cohort, the presence of measurable IAD supports current paediatric hypertension guidelines recommending bilateral BP assessment during initial screening [5]. This is particularly relevant given evidence that single-arm measurements may underestimate BP and delay early identification of cardiovascular risk.

### 4.2. Limb Dominance and Physiological Asymmetry

The finding of higher SBP and HGS values in the dominant limb is consistent with previous literature demonstrating functional asymmetries driven by habitual use and sport-specific demands [31,32,33]. In youth athletes, repetitive unilateral loading may enhance neuromuscular efficiency and induce localized vascular adaptations.

From a mechanistic perspective, increased muscle mass and capillary density in the dominant limb may alter peripheral resistance and blood flow dynamics, contributing to higher SBP readings [34]. This aligns with recent evidence highlighting the interaction between muscular strength and anthropometric characteristics during growth and maturation, which may influence strength expression and overall physiological responses in youth populations [35].

However, the predominance of right-handed participants in this study may have accentuated these findings. Future studies should consider stratification by limb dominance and sport type to better isolate these effects.

### 4.3. Association Between Handgrip Strength and Blood Pressure

The present findings demonstrate moderate positive associations between HGS and SBP in the total sample, with stronger relationships observed in the dominant limb. These results are consistent with previous paediatric studies suggesting that greater muscular strength is associated with higher SBP during growth and development [36,37].

Importantly, these associations were no longer significant after adjustment for anthropometric variables, indicating that body size, rather than muscular strength per se, may primarily drive this relationship. This pattern remained evident after inclusion of age and sex in the adjusted model. This finding suggests that HGS may act as a proxy for overall body mass and lean tissue, both of which are known to influence cardiac output and haemodynamic load [16,38].

From a physiological perspective, children with greater muscle mass may exhibit higher stroke volume and cardiac output, leading to elevated SBP within normal adaptive ranges [38]. Therefore, the positive HGS–SBP relationship observed in unadjusted analyses likely reflects growth-related adaptations rather than pathological cardiovascular mechanisms.

These findings challenge the traditional view that higher physical fitness is uniformly protective, highlighting instead the complex interaction between growth, body composition, and cardiovascular function. Recent evidence supports this interpretation, demonstrating that physical fitness components, including muscular strength and cardiorespiratory fitness, are closely associated with cardiometabolic profiles in youth populations [12,39].

The weaker and inconsistent associations between HGS and DBP observed in this study are also in line with current evidence, as DBP is more closely related to peripheral vascular resistance and autonomic regulation, which may be less directly influenced by muscular strength [34].

### 4.4. Sex Differences

A key finding of this study is the presence of sex-specific differences, with significant associations between HGS and BP observed only in boys. This is consistent with existing literature indicating that boys experience greater increases in muscle mass and strength during maturation, largely driven by androgenic hormonal influences, particularly testosterone [38]. These developmental changes may amplify the relationship between muscular strength and SBP.

The absence of significant associations in girls may reflect differences in body composition, maturational timing, and neuromuscular development. In the present study, the younger mean age of girls may have further attenuated these relationships, as strength and cardiovascular adaptations are typically less pronounced at earlier developmental stages.

However, these sex-specific interpretations should be considered with caution in the absence of direct assessment of biological maturation. Importantly, age was included as a covariate in the multivariable regression analysis; therefore, the reported associations are adjusted for age-related effects. Given the wide age range of participants and the known differences in pubertal timing between boys and girls, maturation status may substantially contribute to the observed sex differences in both HGS and BP. Boys in the present sample were also older and had higher anthropometric values, suggesting that part of the observed sex effect may reflect maturational rather than purely biological sex differences.

Additionally, sex differences in physical fitness characteristics and movement patterns have been shown to influence both musculoskeletal and cardiovascular outcomes in youth athletes [40]. Variations in neuromuscular control, training exposure, and sport-specific demands may further contribute to the observed sex-specific variability.

Taken together, these findings highlight the importance of considering sex as a biological modifier when examining the relationship between muscular strength and cardiovascular function in paediatric populations.

### 4.5. Role of Anthropometric Characteristics

Anthropometric variables emerged as key determinants of SBP in this study, consistent with extensive literature linking body size and composition to BP during growth [3,41]. Height and weight are known to influence cardiac output and vascular dimensions, while BMI and waist circumference reflect adiposity and overall metabolic load.

Importantly, BMI was the only significant predictor of SBP in the regression analysis, reinforcing the central role of body composition in determining BP during childhood and adolescence. This finding aligns with previous evidence demonstrating strong associations between adiposity and elevated SBP in youth populations [3,12,41].

It should also be noted that biological maturation may partly underline the observed associations between anthropometric characteristics and SBP, as pubertal development is closely linked to changes in body size, body composition, and hemodynamic regulation. The absence of maturation assessment limits the ability to disentangle growth- and maturation-related effects from independent anthropometric influences.

The lack of an independent association between HGS and SBP after adjustment suggests that anthropometric factors may confound the relationship between muscular strength and BP. In this context, HGS should be interpreted cautiously, as it reflects not only neuromuscular fitness but also overall body size and lean mass.

Recent studies further support this interpretation, indicating that both fatness and fitness contribute to cardiometabolic risk profiles in children, while sedentary behaviour may additionally modulate these relationships, even in physically active populations [12,39].

The stronger predictive value of anthropometric variables for SBP compared to DBP further supports the notion that systolic pressure is more sensitive to structural and growth-related factors, whereas diastolic pressure is more closely associated with peripheral vascular resistance and regulatory mechanisms.

Taken together, these findings suggest that SBP in youth athletes is more strongly influenced by structural and growth-related characteristics than by functional strength alone.

### 4.6. Inter-Arm Differences and Muscular Asymmetry

The presence of IAD in five (12.2%) participants confirms that this phenomenon is detectable even in young, physically active populations. Although the magnitude of these differences was generally small, the identification of clinically relevant IAD values supports the need for bilateral BP assessment in paediatric settings.

While both SBP and HGS were higher in the dominant limb, their weak association at the inter-limb level suggests that IAD is not solely driven by muscular asymmetry. Instead, vascular factors appear to play a more prominent role in explaining these differences.

Specifically, variations in arterial structure, vascular tone, endothelial function, and regional haemodynamics may contribute to inter-arm differences in BP [25]. These mechanisms are consistent with previous research indicating that anatomical and functional vascular characteristics, rather than musculoskeletal factors alone, underlie IAD.

Taken together, these findings highlight the multifactorial nature of IAD and underscore the importance of comprehensive assessment approaches that consider both vascular and musculoskeletal components.

### 4.7. Strengths

This study has several strengths. It includes bilateral BP assessment, which is not routinely implemented in paediatric populations, allowing the detection of inter-arm differences that may otherwise remain unnoticed. Additionally, the combined evaluation of cardiovascular (BP) and neuromuscular (HGS) parameters provides a more comprehensive assessment of functional status in youth athletes. The use of standardized and validated measurement protocols for both BP and HGS further enhances the methodological rigor and reliability of the findings. Finally, the inclusion of a real-world cohort of non-professional youth athletes increases the ecological validity of the study.

### 4.8. Limitations

This study has several limitations that should be acknowledged when interpreting the findings. First, the relatively small sample size (*n* = 41) limits statistical power, particularly for multivariable analyses, and reduces the generalisability of the results. In addition, the wide age range (6–16 years) introduces substantial developmental heterogeneity that was not stratified in the analysis, which may have influenced both cardiovascular and neuromuscular outcomes. This heterogeneity also implies potential differences in biological maturation between participants, which could not be directly assessed and may confound the observed associations.

Given the small sample size and the number of predictors included in the regression model, these analyses should be considered exploratory and interpreted with caution due to potential limitations in model stability. In addition, only right-arm SBP was included in the multivariable regression model; therefore, potential associations involving left-arm and DBP measures were not explored within the adjusted analytical framework.

Importantly, no direct assessment of biological maturation (e.g., Tanner staging or peak height velocity estimation) was available. Given the broad age range of the sample (6–16 years), puberty-related hormonal and growth changes may substantially influence body composition, muscular strength, vascular responses, and BP. Therefore, biological maturation represents an important potential confounding factor in the present analyses. In particular, the observed sex-related differences in HGS, inter-limb asymmetry, and BP may partly reflect differences in maturation status, especially since boys in the sample were older and presented higher anthropometric values than girls. Consequently, the sex-specific findings should be interpreted with caution.

Participants were recruited from different sport disciplines (rhythmic gymnastics for girls and basketball for boys), which may introduce sport-specific physiological and neuromuscular adaptations that could confound sex-based comparisons in muscular strength and inter-limb asymmetry. Furthermore, training load and sport-specific exposure were not quantified, which may also have contributed to variability in both HGS and BP measures. The cross-sectional design precludes any causal inference regarding the relationships between inter-arm BP differences, muscular strength, and anthropometric characteristics.

Additionally, the predominance of right-handed participants may have introduced bias in inter-limb comparisons and may limit the interpretation of dominance-related findings. Finally, although standardised protocols were used for all measurements, residual measurement variability cannot be excluded.

Overall, these findings should be interpreted as exploratory and hypothesis-generating, and future longitudinal studies incorporating direct measures of maturation and training load are warranted.

### 4.9. Future Directions

Future research should adopt longitudinal designs to better understand how IAD, HGS, and BP evolve throughout growth and maturation. They should incorporate direct maturation assessment and/or age-stratified analyses to better isolate the independent effects of sex and muscular strength on BP outcomes. The integration of additional parameters, including cardiorespiratory fitness, physical activity levels, and sedentary behaviour, would provide a more comprehensive understanding of the underlying mechanisms.

Furthermore, investigating the prognostic value of IAD during childhood for future cardiovascular outcomes represents an important and currently underexplored area of research.

## 5. Clinical Implications

The present findings may have potential clinical relevance for paediatric cardiovascular assessment in physically active youth populations. In this exploratory sample, measurable inter-arm BP differences were identified in a subset of participants, supporting consideration of bilateral BP measurements during initial cardiovascular evaluation in children and adolescents.

Additionally, although HGS demonstrated positive associations with SBP in unadjusted analyses, these relationships were attenuated after adjustment for anthropometric variables. Therefore, HGS assessment may provide complementary information regarding physical and functional characteristics, but its interpretation should account for body size and maturation-related factors rather than being considered an independent cardiovascular marker.

The observed association between BMI and SBP further highlights the importance of monitoring anthropometric characteristics even in physically active youth populations. However, given the exploratory nature of the study and the limited sample size, larger longitudinal investigations are required before these findings can be translated into screening recommendations or broader clinical applications.

## 6. Conclusions

This study identified measurable inter-arm BP differences in a subset of non-professional youth athletes, with five (12.2%) participants demonstrating a systolic inter-arm difference ≥ 10 mmHg. Both SBP and HGS tended to be higher in the dominant upper limb, suggesting the presence of functional and physiological asymmetries in this population.

HGS was positively associated with SBP in unadjusted analyses, particularly among boys; however, these associations were attenuated after adjustment for anthropometric variables, indicating that body size and growth-related factors likely play a key role in this relationship. Among the variables examined, BMI emerged as the most consistent predictor of systolic blood pressure.

Overall, these findings suggest that the relationship between muscular strength, BP, and IAD in youth athletes is complex and influenced by growth and body composition. While bilateral BP assessment and HGS measurement may provide complementary information in paediatric evaluations, the present findings should be considered exploratory. Further longitudinal studies incorporating direct assessment of biological maturation and training load are required to clarify underlying mechanisms and potential clinical relevance.

## Figures and Tables

**Table 1:** Participant Demographics and Anthropometrics by Sex.

Variable	Girls (*n* = 16)	Boys (*n* = 25)	*p*-Value
Age (years)	9.63 ± 2.25	11.96 ± 0.89	<0.001
Height (cm)	139.2 ± 12.5	156.4 ± 9.1	<0.001
Weight (kg)	35.3 ± 10.3	52.2 ± 11.1	<0.001
BMI (kg/m^2^)	17.8 ± 2.6	21.1 ± 2.8	0.002
Waist circumference (cm)	63.0 ± 5.3	73.2 ± 6.6	<0.001

**Table 2:** BP and HGS measurements by gender.

Variable	Girls (*n* = 16)	Boys (*n* = 25)	*p*-Value
**Blood Pressure**			
SBP–Left arm (mmHg)	107.0 ± 11.0	109.1 ± 11.7	0.411
SBP–Right arm (mmHg)	109.0 ± 9.6	110.6 ± 10.0	0.502
DBP–Left arm (mmHg)	66.1 ± 7.6	66.7 ± 7.2	0.786
DBP–Right arm (mmHg)	66.6 ± 6.5	66.5 ± 6.5	0.968
ΔSBP (mmHg)	2.0 ± 1.6	1.5 ± 1.7	0.290
ΔDBP (mmHg)	0.5 ± 0.7	−0.2 ± 0.9	0.073
**Handgrip Strength**			
HGS–Left hand (kg)	17.9 ± 5.3	20.7 ± 6.8	0.171
HGS–Right hand (kg)	19.2 ± 5.6	22.1 ± 6.8	0.149
ΔHGS (kg)	1.3 ± 1.2	2.6 ± 1.7	0.009

SBP = systolic blood pressure; DBP = diastolic blood pressure; HGS = handgrip strength; Δ = interlimb difference (dominant−non-dominant).

**Table 3:** Correlations between HGS and BP.

Variable	Boys r	Boys *p*	Girls r	Girls *p*	Total Sample r	Total Sample *p*
HGS Right–SBP Right	0.596	0.002	0.136	0.616	0.548	<0.001
HGS Right–DBP Right	0.514	0.009	−0.247	0.357	0.316	0.044
HGS Left–SBP Left	0.365	0.073	0.142	0.601	0.457	0.003
HGS Left–DBP Left	0.160	0.446	−0.111	0.682	0.244	0.124

**Table 4:** Multiple linear regression analysis for SBP (right arm).

Predictor	B	SE	*p*-Value	95.0% Confidence Interval for B	
				Lower Bound	Upper Bound
Constant	70.198	9.058	<0.001	51.829	88.568
HGS Right (kg)	0.457	0.280	0.111	−0.111	1.024
Age (years)	−0.506	0.954	0.600	−2.441	1.430
BMI (kg/m^2^)	1.423	0.462	0.004	0.487	2.360
Sex	4.675	2.972	0.124	−1.353	10.703

**Table 5:** Model summary of multiple linear regression analysis for SBP (right arm).

Model	R	R Square	Adjusted R Square
	0.751 ^a^	0.564	0.515

^a^. Predictors: (Constant), Sex, HGS-Right, BMI, Age.

## Data Availability

The data presented in this study are available on request from the corresponding author due to privacy, ethical, and institutional data protection restrictions, in accordance with GDPR (EU 2016/679) and university data protection policies.

## Abbreviations

The following abbreviations are used in this manuscript:

IAD	Inter-arm blood pressure difference
HGS	Handgrip strength
BP	Blood pressure
CVD	Cardiovascular disease
ASHT	American Society of Hand Therapists
WHO	World Health Organization
BMI	Body mass index
SBP	Systolic blood pressure
DBP	Diastolic blood pressure
ΔSBP	Inter-arm differences in SBP
ΔDBP	Inter-arm differences in DBP
ΔHGS	Inter-arm difference in HGS
